# Mesenchymal stem cells secretome-induced axonal outgrowth is mediated by BDNF

**DOI:** 10.1038/s41598-017-03592-1

**Published:** 2017-06-23

**Authors:** Luís F. Martins, Rui O. Costa, Joana R. Pedro, Paulo Aguiar, Sofia C. Serra, Fabio G. Teixeira, Nuno Sousa, António J. Salgado, Ramiro D. Almeida

**Affiliations:** 1 CNC - Center for Neuroscience and Cell Biology, University of Coimbra, Coimbra, Portugal; 2 PhD programme in Experimental Biology and Biomedicine (PDBEB), Center for Neuroscience and Cell Biology, University of Coimbra, Coimbra, Portugal; 3 INEB - Instituto de Engenharia Biomédica, i3S - Instituto de Investigação e Inovação em Saúde, Porto, Portugal; 4 Life and Health Sciences Research Institute (ICVS), School of Medicine, University of Minho, Braga, Portugal; 5 ICVS/3B’s-PT Government Associate Laboratory, Braga/Guimarães, Portugal; 6 School of Health, Polytechnic of Porto (ESS-IPP), Porto, Portugal; 7 Institute for Interdisciplinary Research, University of Coimbra, Coimbra, Portugal

## Abstract

Mesenchymal stem cells (MSCs) have been used for cell-based therapies in regenerative medicine, with increasing importance in central and peripheral nervous system repair. However, MSCs grafting present disadvantages, such as, a high number of cells required for transplantation and low survival rate when transplanted into the central nervous system (CNS). In line with this, MSCs secretome which present on its composition a wide range of molecules (neurotrophins, cytokines) and microvesicles, can be a solution to surpass these problems. However, the effect of MSCs secretome in axonal elongation is poorly understood. In this study, we demonstrate that application of MSCs secretome to both rat cortical and hippocampal neurons induces an increase in axonal length. In addition, we show that this growth effect is axonal intrinsic with no contribution from the cell body. To further understand which are the molecules required for secretome-induced axonal outgrowth effect, we depleted brain-derived neurotrophic factor (BDNF) from the secretome. Our results show that in the absence of BDNF, secretome-induced axonal elongation effect is lost and that axons present a reduced axonal growth rate. Altogether, our results demonstrate that MSCs secretome is able to promote axonal outgrowth in CNS neurons and this effect is mediated by BDNF.

## Introduction

Mesenchymal stem cells (MSCs) can be isolated from a variety of adult tissues, including bone marrow, dental tissue, adipose tissue and Wharton’s jelly of the umbilical cord^1, 2^. Due to these features, the interest on MSCs has been increasing for the last years, with a considerable focus on the use of these cells for cell-based therapies in regenerative medicine.

MSCs have been of particular importance in central and peripheral nervous system repair, due to their regenerative effects^3–5^. The study of regenerative proprieties of MSCs on spinal cord injury has substantially increased recently. These studies were essentially performed with the transplant of MSCs to sites of spinal cord injury and, interestingly, reported that the factors released by MSCs are crucial for spinal cord recovery after an injury^5–9^. In line with these observations, recent studies have shown that most of the regenerative properties of MSCs in nervous system are due to the expression and release of a wide range of molecules (neurotrophins and cytokines) and microvesicles, rather than the ability to differentiate into neuronal or glial cells^10–12^. Thus, administration of MSCs secretome into spinal cord injury sites can be used as an alternative to the grafting of stem cells.

Recent studies have demonstrated that MSCs secretomes are involved in neuronal survival, however, much less is known about their effect on neurite outgrowth. An important observation come from Crigler and colleagues that taking advantage of a co-culture system between MSCs and a neuroblastoma cell line, demonstrated that the molecules released by the MSCs promote cell survival and neurite outgrowth. Moreover, on co-cultures between MSCs and dorsal root ganglia (DRGs), the authors observed similarly neurite outgrowth and also numerous bifurcations in those neurites^10^. In a recent study, Pires and colleagues have demonstrated that the secretome of bone marrow and Wharton jelly derived mesenchymal stem cells induces neurite outgrowth in a neuroblastoma cell line^13^. Moreover, adipose tissue stroma (ASC) secretome induces neurite formation in cells and this effect is mainly regulated by nerve growth factor (NGF)^14^. Altogether, these studies point for the potential importance of MSCs secretome in axonal regenerative therapies.

However, until now the effect of MSCs secretome on axonal development and regeneration in central nervous system (CNS) is still poorly understood. In this work we aimed to uncover the effects of the secretome of a population of mesenchymal progenitors residing in the Wharton Jelly of the umbilical cord, known as human umbilical cord perivascular cells (HUCPVC)^15^, on axonal elongation of CNS neurons. Our results demonstrated that the HUCPVC secretome promotes axonal outgrowth in primary cultures of rat embryonic hippocampal and cortical neurons. Furthermore, we observed a robust axonal outgrowth, when HUCPVC secretome is exclusively applied to distal axons. We also found that brain-derived neurotrophic factor (BDNF) is the key molecule responsible for the observed effect. Together these results show that HUCPVC secretome may be a new approach to modulate axonal outgrowth of CNS neurons.

## Results

### HUCPVC Conditionated Media (CM) induces axonal growth in CNS neurons

**Figure 1:**
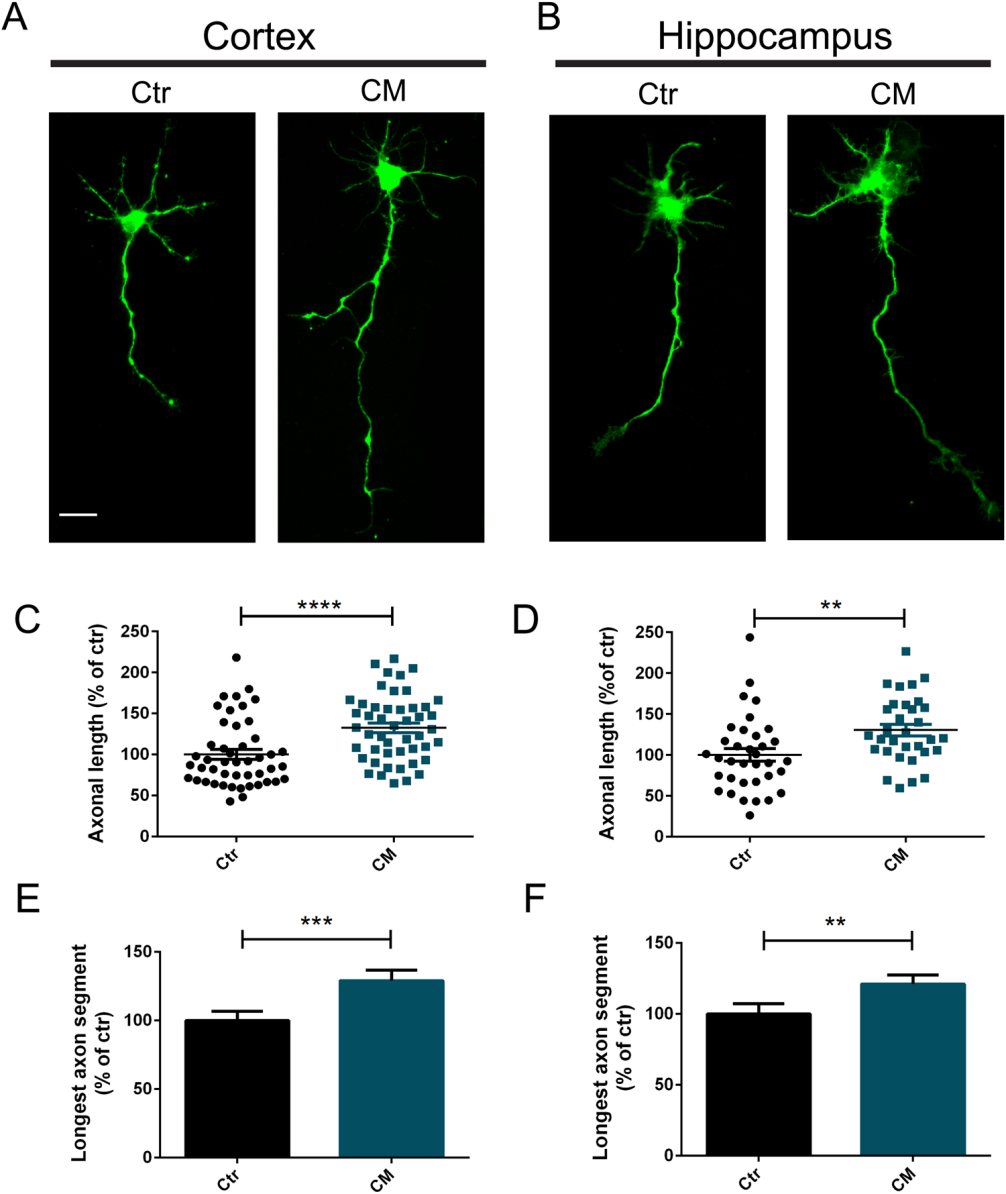
HUCPVC Conditioned Media (CM) induces axonal growth in CNS neurons. (**A**,**B**) Effect of CM in axonal outgrowth on cortical and hippocampal neurons. At DIV3 neurons were stimulated for 14 hours with CM. Axonal outgrowth was assessed by immunocytochemistry using an antibody against Tau, an axonal specific marker. Images were taken from random neurons using an AxioObserver Z1 fluorescent microscope with a PlanApochromat 20× objective. (**C**–**F**) Quantification of axonal length and axonal longest segment. Results show that axonal network increase after 14 h of CM stimulation (**C**,**D**), and in addition neurons stimulated with CM have the longest axonal segments (**E**,**F**), demonstrating that global application of CM to both hippocampal and cortical neurons induce an increase in axonal outgrowth. Axonal length and longest axonal segments analysis was performed with Image J 1.45e software. Bars and plots represent the mean ± SEM of approximately 45 neurons randomly selected of 3 independent experiments. (**C**) ****Represents p < 0.0001; (**D**) *Represents p = 0.0025; (**E**) ***Represents p = 0.0010; (**F**) *Represents p = 0.0083 by Mann Whitney unpaired t-test when compared to Ctr. The scale bar is 25 µm.

We first asked if CM is able to induce axonal outgrowth in CNS neurons. To test this possibility, we used rat embryonic cortical (Fig. 1A) and hippocampal (Fig. 1B) neurons. At day *in vitro* (DIV) 3, cell medium was removed and CM was applied to the neuronal cultures for 14 hours. To control for possible non-specific effects of media exchange, in the control condition (Ctr) media was also replaced by fresh neurobasal medium (NBM). Axons were stained against the axonal marker Tau and both axon length (Fig. 1C,D) and axonal longest segments (Fig. 1E,F) were evaluated by immunocytochemistry. It was observed that, in the presence of CM, axonal outgrowth significantly increased 132.50% ± 5.82 and 130.30% ± 7.15 in cortical and hippocampal axons, respectively, when compared to control. In addition, axons treated with CM presented the longest axon segments when compared to control axons (129.00% ± 7.60 in cortical axons and 120.90% ± 6.58 in hippocampal axons) (Fig. 1). These observations demonstrate that CM administration enhances axonal outgrowth in developing CNS neurons.

### Axonal-specific stimulation with CM induces axonal growth of CNS neurons

**Figure 2:**
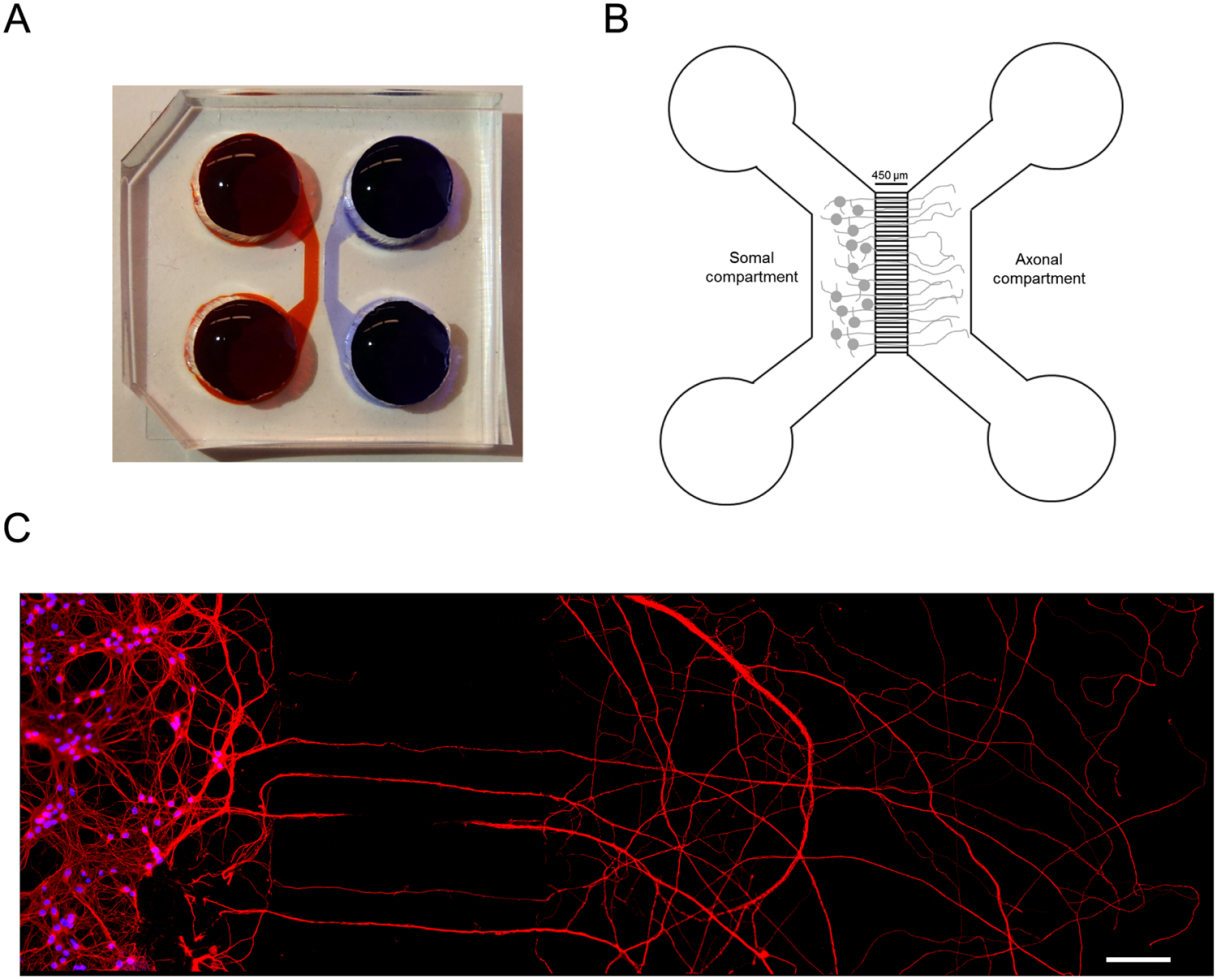
Microfluidic chambers for culturing CNS neurons. (**A**,**B**) Representative model of a microfluidic chamber. These small systems (20 mm × 25 mm) consist of a molded PDMS chamber placed against a glass coverslip. The microfluidic device consist of a somal compartment (red) and an axonal compartment (blue), each 1.5 mm wide, 7 mm long, which are separated by microgrooves (450 μm long, 10 μm wide). Neurons are plated in the somal compartment and between days 4–5 the axons pass through the microgrooves into the axonal compartment. The height difference between microgrooves (3 μm) and compartments (100 μm) combined with a minimal volume difference between the two sides (~25 μl) leads to a fluidic isolation between the two compartments. (**C**) Representative image of cortical neurons cultured in microfluidic chambers. At DIV5-6, cortical neurons were immunostained for tubulin (red) and stained for DNA (blue). The image shows that cell bodies are restricted to the somal compartment while in the opposite compartment only axons are observed. Thus microfluidic chambers allow axonal isolation and specific manipulation of distal axons without soma contribution (for further details see the Material and methods section and ref. 17). Contiguous images were taken from a random area of the microfluidic chamber using an AxioObserver Z1 fluorescent microscope with a PlanApochromat 20× objective and assembled into a single image using the ZEN 2011 software. The scale bar is 100 µm.

**Figure 3:**
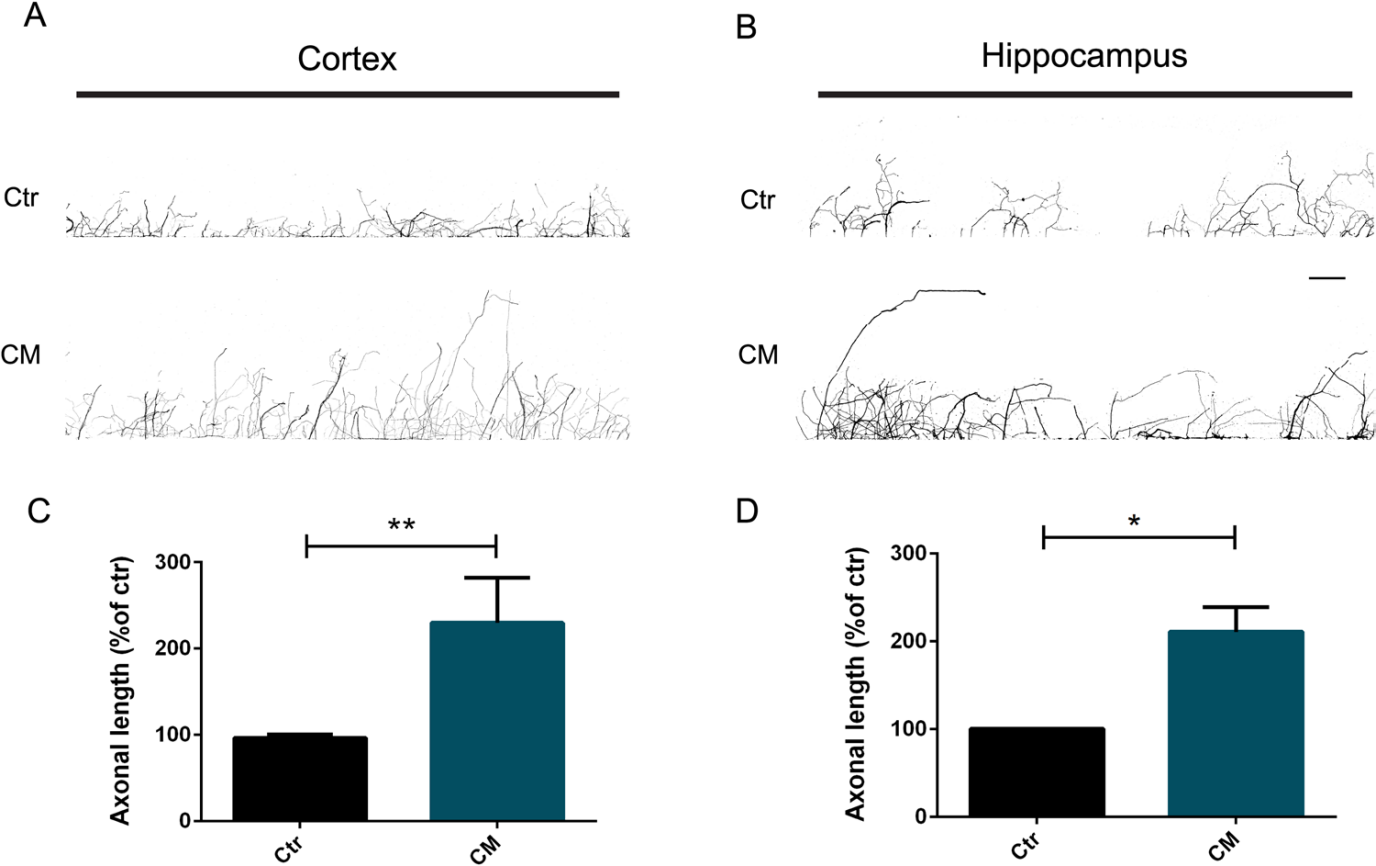
Axonal-specific stimulation with CM induces axonal growth of CNS neurons. (**A**,**B**) Effect of local application of CM on axonal outgrowth. Cortical (**A**) and hippocampal (**B**) axons present in the axonal compartment were stimulated with CM for 24 h at DIV5-6. Axonal outgrowth was evaluated by immunocytochemistry using anti-tubulin βIII. The area selected comprises 3 mm of chamber length which comprises the area between the first and the last microgrooves. Contiguous images were taken using an AxioObserver Z1 fluorescent microscope with a PlanApochromat 20× objective and assembled into a single image using the ZEN 2011 software. (**C**,**D**) Quantification of axonal length. Results show that in the presence of CM the axonal network significantly increases comparatively to control, demonstrating that specific local application of CM to cortical and hippocampal axons promotes axonal outgrowth. Axonal network was measured using Neurolucida software. Bars represent the mean ± SEM of 3 independent experiments. *Represents p = 0.0286 by Mann Whitney unpaired t-test when compared to Ctr. (**C**) **Represents p = 0.0079 by Mann Whitney unpaired t-test when compared to Ctr. The scale bar is 250 µm.

We then sought to evaluate if CM applied exclusively to axons affects axonal outgrowth. In order to recapitulate the physiological environment more closely, we used microfluidic chambers that allow the physic and fluidic separation of axons from soma and dendrites^16–18^. These microfluidic devices are composed of a molded poly-dimethylsiloxane (PDMS) piece placed against a glass coverslip (Fig. 2A). The devices used in this work have two compartments, the somal and the axonal, which are connected through a set of small channels, the microgrooves (Fig. 2B). This structure confines cell bodies in the somal compartment but allows axons to grow through the microgrooves into the axonal compartment. Due to the higher growth rate and length of axons when compared to dendrites, only axons cross the microgrooves into the axonal compartment, since dendrites are not long enough to go through the 450 μm long microgrooves (Fig. 3C). Besides physical isolation, microfluidic devices also feature fluidic isolation that is accomplished by a minimal volume difference between the somal and the axonal compartment. This slight volume difference accompanied by the high fluidic resistance of the microgrooves allows the fluidic isolation of the axonal compartment^18, 19^.

Cortical and hippocampal neurons were grown in microfluidic chambers until DIV5, next CM was applied for 24 h only to the axonal compartment; fresh NBM was added in the control conditions. In the presence of CM, both cortical (209.50% ± 52.23) (Fig. 3A) and hippocampal (210.70% ± 28.12) (Fig. 3B) axonal network was increased when compared to control. We also observed that CM has a prominent effect in cortical axons, where we observed a more intricate axonal network (Fig. 3). Thus, these observations demonstrate that local administration of CM to distal axons is sufficient to induced robust axonal outgrowth.

### BDNF is an important molecule for CM-induced axonal outgrowth in cortical neurons

**Figure 4:**
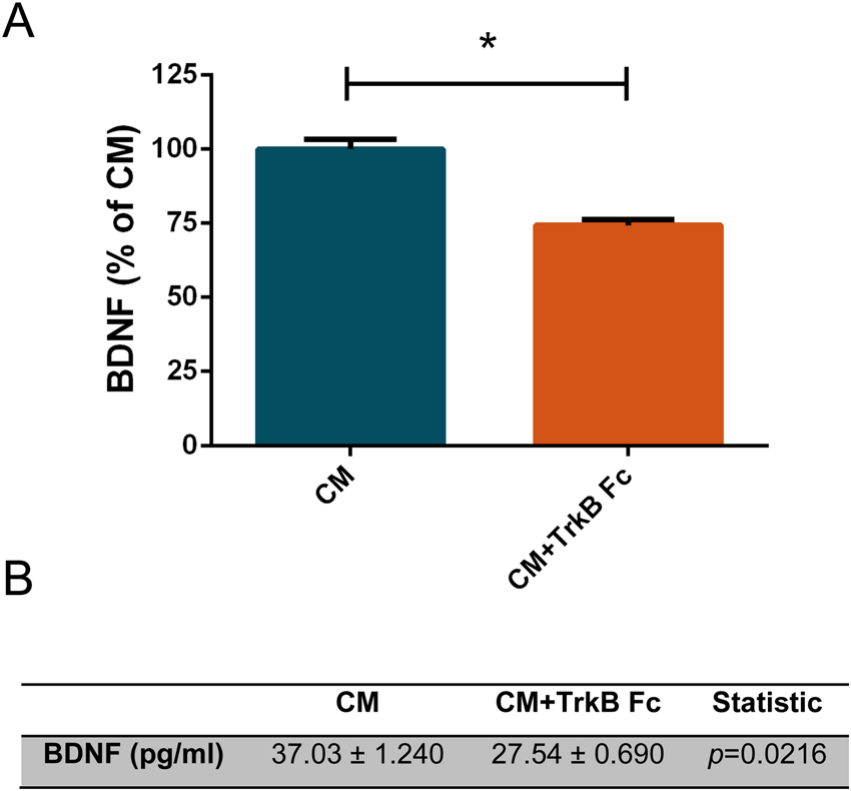
TrkB Fc reduces the levels of BDNF present in CM. (**A**) HUCPVC conditioned media was incubated with TrkB Fc and the levels of BDNF were determined by the Bioplex-Luminex assay. Our results show that TrkB Fc is able to cause a reduction of approximately 25% in the levels of BDNF in CM (74.37 ± 1.86), comparatively to CM (100.0 ± 3.35) proving that TrkB Fc is able to significantly deplete BDNF from CM. (**B**) BDNF concentration in CM is 37 pg/ml. On the other hand, the presence of TrkB Fc, reduces the levels of BDNF present in CM to approximately 27 pg/ml. BDNF levels were measured using a MILLIPLEX Kit. *Represents p = 0.0216 by Mann Whitney unpaired t-test when compared to CM.

**Figure 5:**
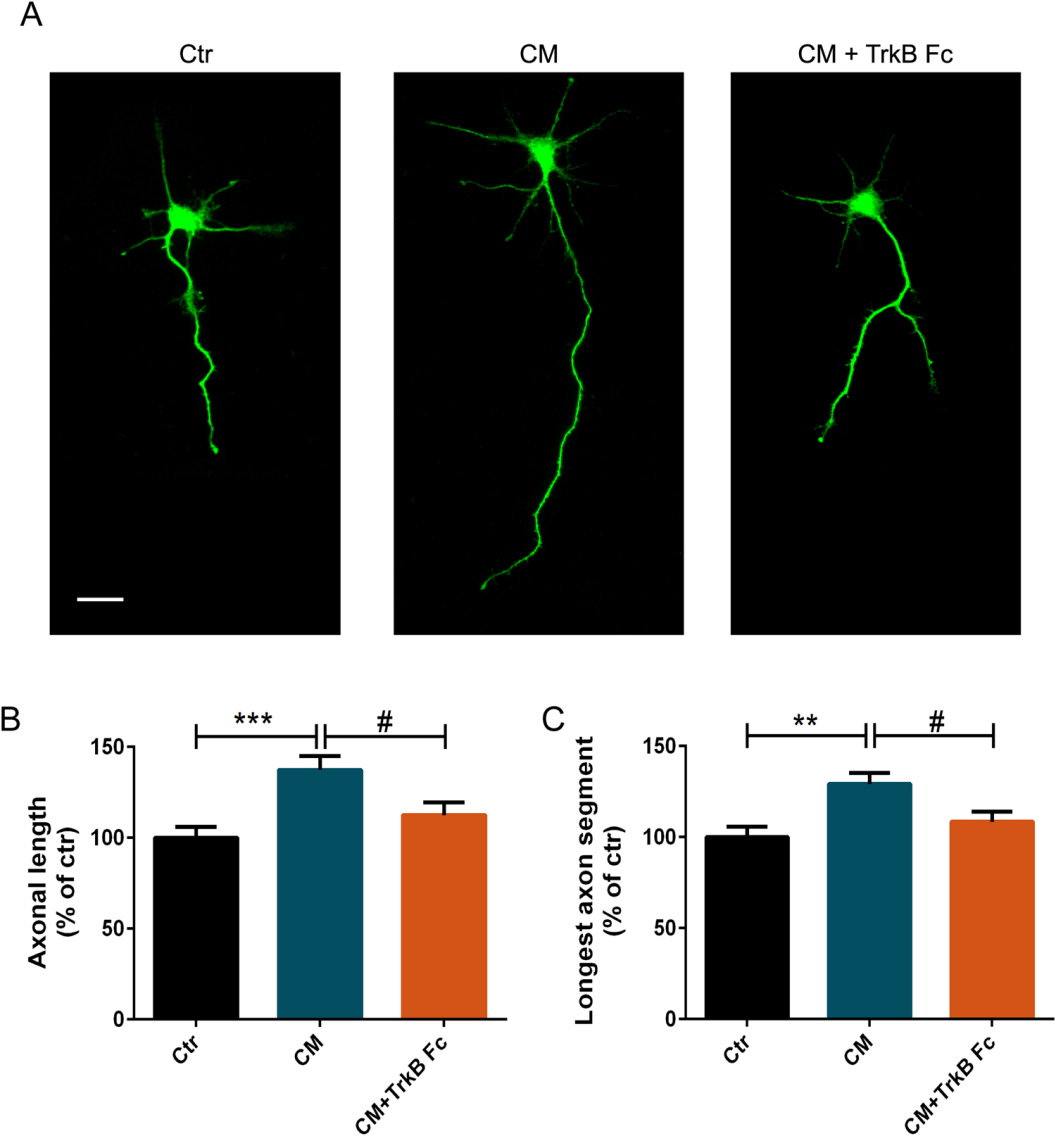
BDNF is the main component of CM-induced axonal outgrowth in cortical neurons. (**A**) Depletion of BDNF prevented CM-induced axon outgrowth. Cortical neurons were globally stimulated with CM for 14 h at DIV5-6. Axonal outgrowth was assessed by immunocytochemistry using an antibody against Tau. Images were taken from random neurons using an AxioObserver Z1 fluorescent microscope with a PlanApochromat 20× objective. Global application of CM induced axon outgrowth, as previously described. However, when BDNF is depleted from the conditioned media using a specific antibody this effect is abolished. In the absence of BDNF, the axonal length and the range of the longest axons are similar to control values, indicating that BDNF is one the major molecules involved in the CM-induced axonal outgrowth effect. (**B**,**C**) Quantification of axonal length (**B**) and axonal longest segment (**C**). Axonal length and longest axonal segments measuring was performed with Image J 1.45e software. Results show that CM stimulation increases the axonal network, and in addition neurons stimulated with CM have the longest axonal segments. However, when BDNF is depleted, with the addition of 1 µg/mL of TrkB Fc, a molecule that binds specifically to BDNF molecules neutralizing them, there is no increase in axonal length nor in the axonal longest segments. Bars and plots represent the mean ± SEM of approximately 60 neurons randomly selected of 4 independent experiments. (**B**) ***Represents p = 0.0004 by one-way ANOVA analysis of variance using Tukey post test when Ctr is compared to CM; ^#^represents p = 0,0307 by one-way ANOVA analysis of variance using Tukey post test when CM is compared to CM + TrkB Fc. (**C**) **Represents p = 0,0014 by one-way ANOVA analysis of variance using Tukey post test when Ctr is compared to CM; ^#^represents p = 0.0341 by one-way ANOVA analysis of variance using Tukey post test when CM + TrkB Fc compared to CM. The scale bar is 25 µm.

It has been previously shown that the secretome of human MSCs comprises several distinct protein categories, such as neurotrophic factors and cytokines and also microvesicles and exosomes, which might contain genetic material that can be transferred to cells^20^. Within the growth factor family several molecules were identified, namely, vascular endothelial growth factor (VEGF), NGF and insulin-like growth factor 1 (IGF-1). Neurotrophins can regulate axonal growth in distinct sets of neurons^21^ and are therefore a likely candidate to mediate the observed axonal outgrowth. BDNF was one of the neurotrophins identified in the secretome of human MSCs^22, 23^ and is a well described neurite outgrowth inducer^21^. Therefore, we next asked if BDNF could mediate the CM-induced axonal outgrowth effect. In order to address this question we need to deplete BDNF from CM. This was achieved by the addition of TrkB Fc, a molecule that specifically binds and neutralizes BDNF^24^. To demonstrate that TrkB Fc is able to neutralize BDNF from CM, we evaluated the levels of BDNF present in the CM in the presence or absence of TrkB Fc (Fig. 4). Our results demonstrate that BDNF is present in CM in a concentration of approximately 37 pg/ml (Fig. 4), which is in accordance with previous studies that evaluate the levels of BDNF in cortical and hypothalamic neurons^25, 26^. After the administration of TrkB Fc, the levels of BDNF are reduced to approximately 27 pg/ml (Fig. 4), demonstrating that TrkB Fc is able to significantly deplete BDNF from CM. These results are in agreement with previous studies, which demonstrated that TrkB Fc is able to deplete BDNF from the culture media^24, 27, 28^. Moreover, it was also described that administration of TrkB Fc blocked the BDNF-induced phosphorylation of TrkB receptors, demonstrating that TrkB Fc is sufficient to neutralize the action of BDNF^27, 28^. Next, TrkB Fc was added to conditioned media 15 min before media was added to cortical neurons. Remarkably, BDNF depletion from the CM, globally applied to neurons, reduced CM-mediated axonal outgrowth (Fig. 5). The average length of axonal outgrowth in the presence of TrkB Fc was similar to basal levels (112.40% ± 6.96), while axons in contact with non-depleted CM have a significant increase of axonal length (137.30% ± 7.58) (Fig. 5B). Moreover, the absence of BDNF in CM resulted on axonal longest segments with similar levels to control (108.50% ± 5.51) and shorter comparatively to CM-treated axons (129.20% ± 6.14) (Fig. 5C), demonstrating the importance of BDNF on the CM-induced axonal extension.

### BDNF regulates CM-induced axonal outgrowth in distal axons

**Figure 6:**
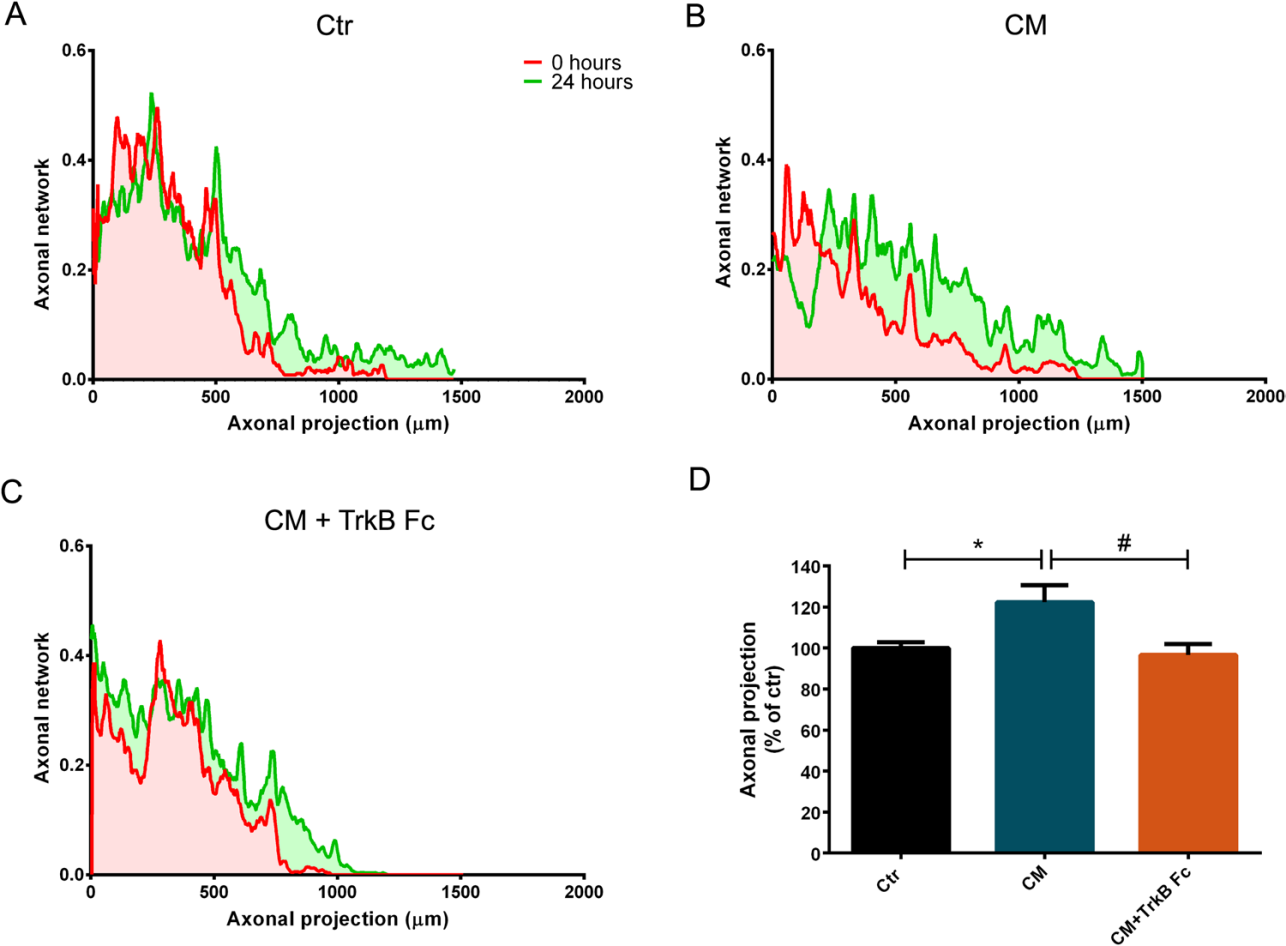
BDNF is the molecule responsible for CM-induced axonal elongation in distal cortical axons. (**A**–**C**) Quantification of axonal outgrowth with the MATLAB script AxoFluidic. Profile along the xx axis of the microfluidic device showing the area occupied by the axons at 0 hours (clear red area) and at 24 hours (clear green area). Red line represents axonal projection at 0 hours and the green line represents axonal projection at 24 hours. (**D**) Quantification of axonal projections outgrowth between 0 and 24 hours. Distal axons treated with CM for 24 hours present more pronounced elongation than control or CM-lacking BDNF axons. Bars represent the mean ± SEM of 6 microfluidic chambers randomly selected of 3 independent experiments. *Represents p = 0.0346 by one-way ANOVA analysis of variance using Tukey post test when Ctr is compared to CM; ^#^represents p = 0.0145 by one-way ANOVA analysis of variance using Tukey post test when CM is compared to CM + TrkB Fc.

We next asked if BDNF could exert its action through a localized effect. As previously, we took advantage of microfluidic chambers to test CM in distal axons, eliminating the contribution of other molecular mechanisms activated by CM in the soma and dendrites. For this purpose, cortical neurons were plated in microfluidic chambers and transduced with a lentivirus expressing enhanced-green fluorescent protein (EGFP) at DIV1, enabling us to analyze axonal outgrowth in live neurons. At DIV5, axons present in the axonal compartment were stimulated with CM or with CM with TrkB Fc for 24 hours. Fresh NBM was added to control axons. Images were acquired just prior to stimulation and 24 hours later. Axonal elongation was analyzed using the AxoFluidic Matlab plugin (see material and methods) (Fig. 6A–C). Notably, the CM-induced axonal outgrowth effect (122.30% ± 8.31) was lost when BDNF was depleted from the media (96.56% ± 5.42) (Fig. 6D), demonstrating that BDNF acts locally to induce axonal outgrowth without contribution from the cell body.

**Figure 7:**
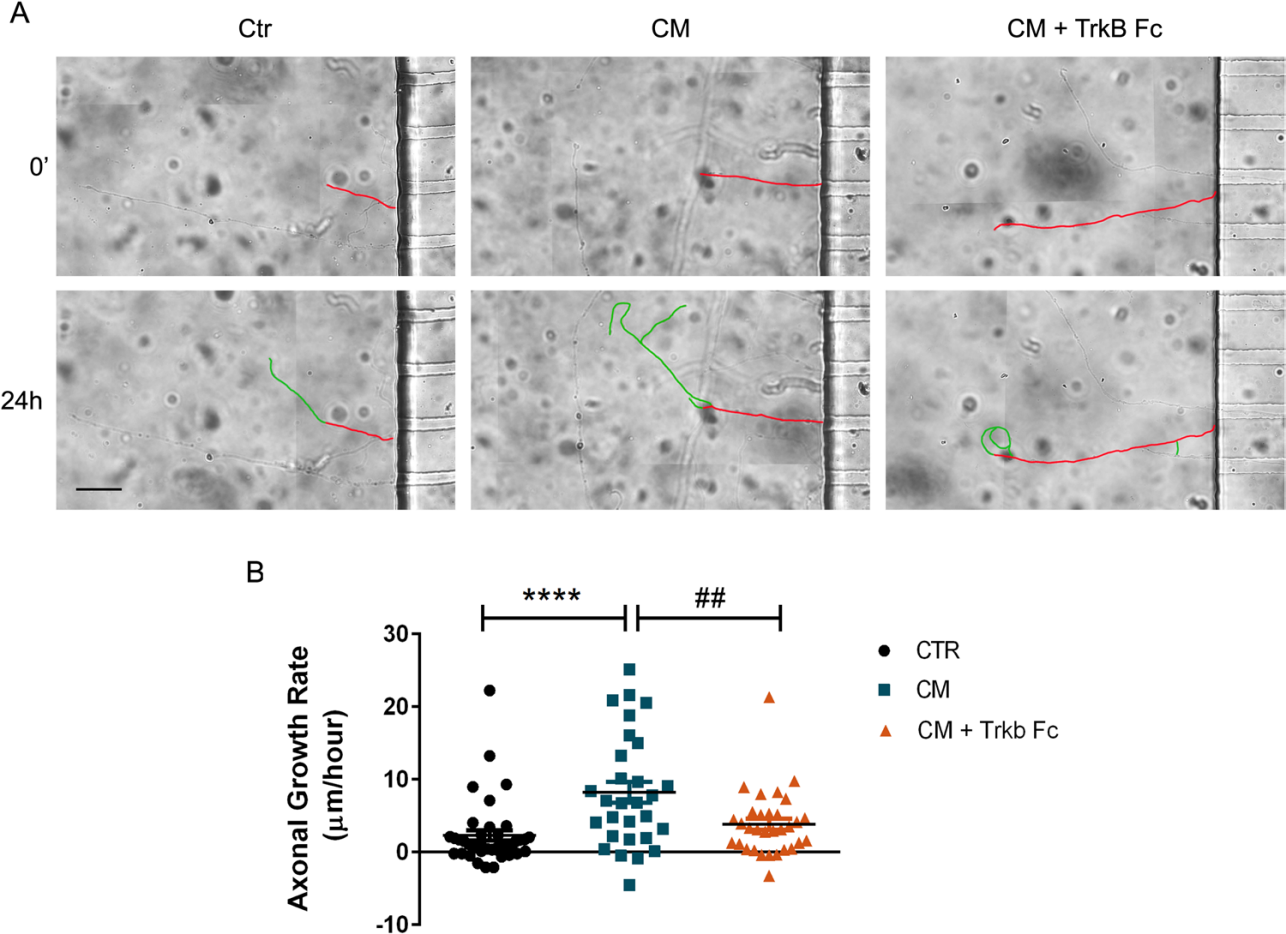
BDNF works as a localized signal in CM-induced axonal outgrowth. (**A**) Depletion of BDNF from CM in distal cortical axons abolished CM-induced axonal outgrowth. The axonal outgrowth of individual axons was assessed by live-cell imaging at the moment of CM addition to the axonal compartment of microfluidic chambers and 24 h later. The results show that BDNF depletion results in the abolishment of CM-induced axonal outgrowth in isolated cortical axons, in agreement with the results observed in Fig. 6, demonstrating that BDNF is a key component of CM-induced axonal elongation. Contiguous images were taken using an AxioObserver Z1 fluorescent microscope with a PlanApochromat 20× objective and assembled into a single image using the ZEN 2011 software. (**B**) Quantification of axonal growth rate. Fresh NBM, CM and CM + Trkb-Fc were added to the axonal compartment at DIV4 and axons allowed to develop for 24 hours. After this period, new images were acquired at the exact same position in the axonal compartment of microfluidic chambers, and the growth rate of individual axons were analyzed with Image J 1.45e software (see Material and methods for details). Results show an increase in axonal growth rate during CM stimulation, however depletion of BDNF blocks the observed axonal growth rate after CM stimulation. Plots represent the mean ± SEM of approximately 30 axons selected of 3 independent experiments. ****Represents p < 0.0001 by one-way ANOVA analysis of variance using Tukey post test when Ctr is compared to CM; ^##^represents p = 0.0080 by one-way ANOVA analysis of variance using Tukey post test when CM is compared to CM + TrkB Fc. The scale bar is 50 µm.

We then sought to calculate the axonal growth rate in live neurons, in which we can evaluate the behavior of individual axons. For this, cortical neurons were cultured in microfluidic chambers and axonal length of individual axons was measured at t = 0 h and t = 24 h using brightfield live imaging (Fig. 7A). CM-treated axons presented an average axonal growth rate of approximately 4 fold higher (8.24 μm/hour ± 1.44) than control-treated axons (2.28 μm/hour ± 0.71), clearly evidencing that CM induces an enhance cells were subjected to a pre-fixation ment in the axonal growth rate. Strikingly, when BDNF was depleted from CM, with TrkB Fc, the axonal growth rate is lower (3.85 μm/hour ± 0.78), presenting similar levels to basal conditions, undoubtedly demonstrating that CM-induced axonal extension is mediated by local BDNF action.

## Discussion

**Figure 8:**
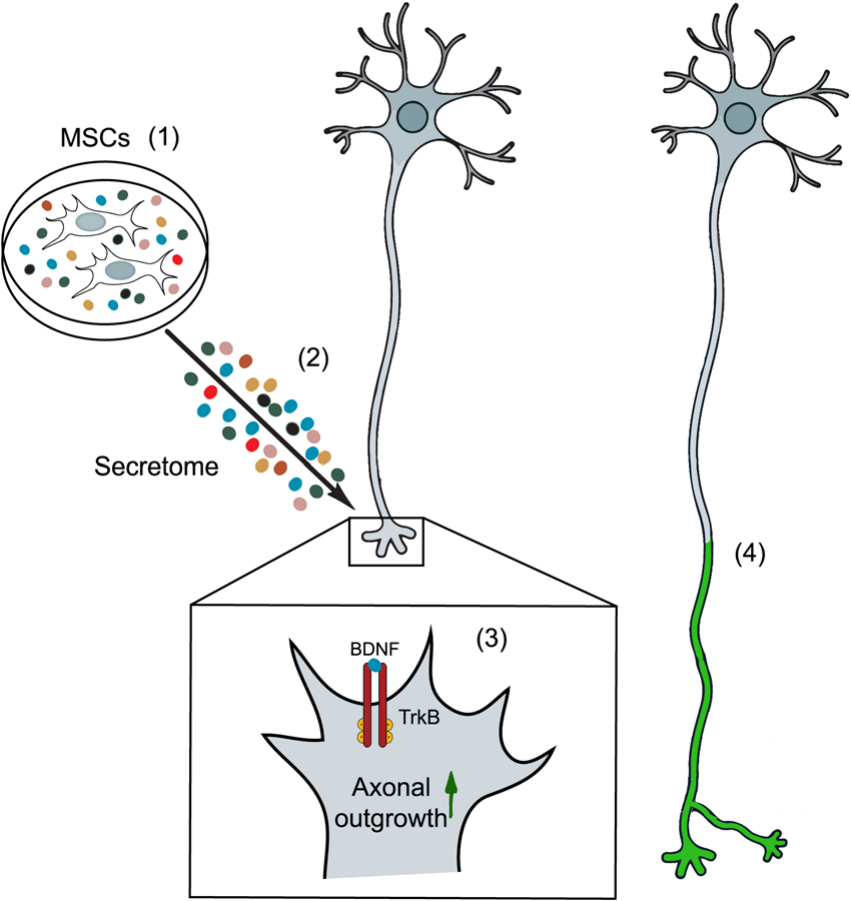
Proposed model for secretome-induced axonal outgrowth. MSCs secretome is composed of a diverse set of molecules, including trophic factors, cytokines, microvesicles and exosomes (1). When the secretome is applied to distal axons (2), BDNF, one of the molecules present, will bind to TrkB present in the membrane of growth cones (3). This interaction leads to the activation of the receptor and downstream signaling pathways, resulting in the activation of the machinery necessary for axonal outgrowth (4). The secretome can act locally, at sites distant from the cell body, engaging intra-axonal mechanisms effectively promoting axonal elongation.

In this study is demonstrated that the HUCPVC secretome, in the form of conditioned media, has the ability to induce axonal outgrowth in central nervous system neurons. To date, several groups have demonstrated that transplantation of MSCs into spinal cord favors nerve regeneration^5–9^. Moreover, recent studies suggest that MSCs secretome promotes neurite outgrowth^10, 13, 29^. However, it remained unknown the effects of mesenchymal stem cells secretome in CNS neurons, specifically in the axons. Our results indicate that CM promotes axonal elongation in cortical and hippocampal neurons (Fig. 1). Importantly, we show that a localized application of the secretome to distal axons induces robust axonal outgrowth (>200%, Fig. 3), which indicates that the secretome is able to locally activate the machinery involved in axonal outgrowth (Fig. 8).

In order to more closely recapitulate the physiological environment of distal axons, we used microfluidic devices (Fig. 2). The axons present in the axonal compartment of these devices were specifically treated with CM, which allowed us to evaluate its effect locally. Notably, CM added only to axons is able to induce axon elongation, expanding the results performed in whole neuronal cultures and suggesting that the set of molecules that comprises CM can act at the axonal level to induce the activation of the axon outgrowth machinery (Fig. 3). Moreover, the local axonal application of CM and consequent enhancement on axonal outgrowth, demonstrates that CM effect is independent of any potential CM-activated mechanisms at the soma. This is of great importance for axonal regeneration, since a CM-based therapeutical approach must rely on a local application to the site of injury and not to entire neuronal cells, which makes this a simpler and accurate strategy to the treatment of dysfunctions that affect distal axons, such as, spinal cord injury.

MSCs secretomes present a high range of molecules, such as cytokines and growth factors^11, 30–32^. BDNF is a neurotrophin that, together with TrkB, its cognate receptor, is present throughout CNS^33^. Moreover, this neurotrophin is involved in axon outgrowth in the CNS^34, 35^. However, the importance of BDNF in secretome-induced axonal outgrowth in CNS is still unknown. Taking this in consideration, we decided to deplete BDNF from CM in order to evaluate the importance of this molecule in CM-induced axonal elongation. Remarkably, when BDNF was removed from CM (Fig. 4), the axonal outgrowth induced by CM applied globally (Fig. 5) or locally (Fig. 6) was lost, suggesting that this neurotrophin has a key role in CM-induced axonal elongation. To further corroborate our results the individual axonal growth rate was also analyzed. Axons treated with CM-depleted BDNF presented a growth rate that did not surpass basal levels (Fig. 7), unequivocally demonstrating that BDNF is the key molecule responsible for CM-induced axonal outgrowth in CNS neurons. Therefore, the manipulation of BDNF levels or the signaling pathways activated by BDNF can be a potential tool for novel therapeutics in axonal regeneration.

HUCPVC secretome present on its composition a wide range of molecules with different properties^31, 32^. Although our data shows a complete reversion of CM-induced axonal outgrowth when BDNF is depleted from the media, we cannot exclude the possibility that other factors present in HUCPVC secretome might also induce axonal outgrowth. In fact, molecules like glia-derived nexin (GDN), pigment epithelium-derived factor (PEDF), IGF-1, VEGF and NGF were found in the secretome of human MSCs^20^. IGF-1 has, in fact, been shown to induce axonal outgrowth in corticospinal motor neurons^36^ and also axonal regeneration in retinal ganglion cells^37^ and is another good candidate to test. Furthermore, VEGF was described as an inductor of axonal outgrowth in different neuronal populations, such as DRG, superior cervical ganglia (SCG) and retinal ganglion cell (RGC) neurons^38, 39^, and can be another potentially candidate to test. Additionally, NGF is a well-known promoter of axonal outgrowth^34^. NGF exerts its effect through activation of TrkA receptors and it was previously demonstrated that NGF present in the secretome of ASC is essential for neurite outgrowth of PC12 cells^14^. We can speculate that these molecules can promote axonal elongation in a synergistic way. However, due to the striking effect of BDNF depletion from HUCPVC conditioned media in axonal outgrowth, the other molecules present in CM might just have a co-adjuvant role in the outgrowth promoted by BDNF. Future studies regarding other molecules promoters of axonal elongation present in CM must be performed in order to clarify the complete mechanism behind CM-induced axonal outgrowth. Nevertheless, based on our results, BDNF can potentially be the best candidate to pursue future therapeutic approaches.

Binding of BDNF to TrkB induces the dimerization and subsequent autophosphorylation of the receptor. Similarly to other receptor tyrosine kinases TrkB can induce the activation of Erk, PI3K-Akt or PLCγ pathways^40–42^. BDNF/TrkB signaling is involved in the regulation of a multiplicity of neuronal development processes such as neuronal differentiation, neuronal survival, axonal and dendritic branching, axonal outgrowth and synaptic plasticity^43^. In line with this, Erk and PI3K-Akt signaling pathways have been linked to axonal outgrowth^44^. Thus, these signaling cascades are important for axonal regeneration mediated by BDNF. In fact, it was observed that TrkB signaling is important for the formation of actin waves, required for axonal re-growth^45^. Thus, in future work it will be very important to identify which signaling pathway(s) downstream of BDNF are activated by CM and regulate axonal outgrowth.

In fact, BDNF is one of the major molecules involved in neuronal development in the nervous system. This neurotrophin was shown to regulate cell survival, synapse formation and axonal outgrowth, and its axonal elongation properties were demonstrated in different neuronal populations^21, 46–49^. Additionally, exogenous administration of BDNF to sites of spinal cord injury has been tested with interesting results, namely, regeneration of forelimb function after cervical lateral hemisection as well as induction of axonal elongation of rubrospinal, reticulospinal, vestibulospinal, raphespinal, and local sensory and motor axons^50^. For example, it was demonstrated that BDNF induces axonal outgrowth of motor, sensory and coerulospinal neurons^51^. Furthermore, Lopatina and colleagues have shown that the adipose-derived mesenchymal stem cells (ASCs) ability to induce motor axons regeneration is dependent on the BDNF secreted by these cells^52^. These observations strongly support our results, reinforcing the idea that BDNF is a key molecule for CM-induced axonal outgrowth (Fig. 8). However, in this work we have used exclusively the CM and, thus, our results demonstrate for the first time that the secretome, through the action of BDNF, is able to potentiate axonal elongation of central nervous system neurons. The use of secretome instead of MSCs, overcomes some problems related with the graft of these cells, such as, the low survival rate when transplanted into the CNS, the requirement of a high number of cells for transplantation and long periods of expansion of MSCs *in vitro* prior to transplantation, which cause phenotypic alterations affecting the secretome. Thus, the secretome can be an advantageous strategy to be used as therapeutic tool in the development of novel solution for the treatment axonal dysfunctions.

Altogether these results demonstrate undoubtedly that HUCPVC secretome promotes axonal outgrowth of CNS neurons and, interestingly, this effect is axonal specific, i.e., application of HUCPVC secretome only to axons promotes axonal elongation. This study provides news insights of the effects of MSCs secretomes in the nervous system. Particularly, we identified BDNF as the molecule responsible for the secretome-induced axonal outgrowth and, thus, advancing our knowledge of MSCs secretome (Fig. 8). On, a broader perspective this work might contribute to bringing us closer for future treatments of spinal cord injury and other CNS diseases.

## Methods

### Primary neuronal cultures

Wistar-Han rats (Charles River, Barcelona, Spain) were housed (two per cage) and maintained in a controlled environment at 22–24 °C with 55% humidity, on a 12 h light/dark cycle and fed with regular rodent’s chow and tap water ad libitum. All manipulations were done after approval from the CNC Animal Welfare Committee (ORBEA; ID: ORBEA 28022013) and from the Portuguese national authority for animal experimentation, Direção Geral de Veterinária (DGAV; ID: DGAV0421), and in accordance with the approved guidelines and regulations on animal care and experimentation stated in the European Union Directive 2010/63/EU. For this study, 17 animals were used. Primary cultures of rat embryonic hippocampal neurons were prepared from the hippocampus of embryonic day 17–18 Wistar rat embryos as described previously^53–55^. After dissection, cortices and hippocampi were treated for 15 min at 37 °C with trypsin (0.045%) and deoxyribonuclease (0.01% v/v) in Hank’s balanced salt solution (HBSS) (5.36 mM KCl, 0.44 mM KH2PO4, 137 mM NaCl, 4.16 mM NaHCO3, 0.34 mM Na2HPO4.2H2O, 5 mM glucose, 1 mM sodium pyruvate, 10 mM HEPES and 0,001% phenol red). Hank’s solution with trypsin was removed and the hippocampi were washed with plating medium containing 10% fetal bovine serum (FBS) to stop trypsin activity. In order to obtain a homogeneous cell suspension the hippocampi were mechanically dissociated with a P1000-pipette and then with a Pasteur pipette. Similar to hippocampus, Hank’s solution with trypsin was removed and the cortices were washed 6 times with HBSS and then mechanically dissociated with a glass pipette, obtaining a homogeneous cell suspesion. Cells were then added to the somal side of microfluidic chambers coated with poly-D-lysine (PDL) (0.1 mg/ml) and laminin (2 μg/ml); or plated at the middle of a coverslip in a 24-well plate. In hippocampus cultures, after 2 h incubation at 37 °C, the plating medium was removed and replaced for culture medium (Neurobasal medium supplemented with 2% B27, 25 μM glutamate, 0.5 mM glutamine and penicillin/streptomycin). In cortical cultures, after 2 h incubation at 37 °C, the plating medium was removed and replaced for cultures medium without glutamate (Neurobasal medium supplemented with 2% B27, 0.5 mM glutamine and penicillin/streptomycin). At DIV 3/4, in both cultures, the mitotic inhibitor 5-Fluoro-2′-deoxyuridine thymidylate synthase inhibitor (5-FDU) in glutamate-free culture medium was added to the cultures at a final concentration of 10 μM to reduce contamination with glial cells.

### Microfluidic devices

Microfluidic devices were prepared as described before^17^. Briefly, a PDMS chamber was assembled onto a glass coverslip. PDMS was prepared from the Sylgard 184 Silicone elastomer kit (Dow Corning), decanted onto the microfluidic molds and cured for 4–6 h.

### Conditioned Media

HUCPVCs were isolated as described previously^32^. HUCPVCs were ressuspended in α-Mem medium (Invitrogen, USA) supplemented with 1% of antibiotic/antimycotic (Invitrogen, USA) and 10% of FBS (Invitrogen, USA) and, subsequently, plated on culture at a density of 4.0 × 10^3^ cells/cm^2^. The cells were maintained at 37 °C, 5% CO_2_, 95% air and 90% relative humidity and the culture medium was changed every 3 days until reach confluency. Secretome was collected in the form of conditioned media (CM) from P3 HUCPVCs. For this purpose cells were plated out at a density of 4000 cells/cm^2^ and allowed to grow for 3 days. Following this, culture medium was renewed (with no addition of exogenous factors) and CM collected 24 hours thereafter (cell culture media was not renewed or added during this time period). For CM collection HUCPVCs were incubated with Neurobasal-A medium. Before cell culture experiments HUCPVCs secretome was 100x concentrated using a Vivaspin 20 sample concentrator (5 kDa; GE Healthcare) by ultracentrifugation at 3000 g for 45 min, as previously described by Teixeira *et al*.^56^.

### Conditioned Media experiments

Culture media was replaced with either fresh Neurobasal media containing penicillin/streptomycin and glutamine or HUCPVCs-conditioned media (see above), for 14 h in whole neuronal cultures experiments or 24 h in local axonal application experiments. For CM and CM + TrkB Fc experiments, the media was supplemented with TrkB Fc (1 µg/ml) (R&D Systems).

### BDNF depletion

BDNF was depleted from conditioned medium using the recombinant Human TrkB Fc chimera protein (#688-TK; R&D Systems). Briefly, Protein A-Agarose (#sc-2001; Santa Cruz Biotechnology, Inc.) was pre-coated with 1 µg/ml TrkB Fc and gently mixed with conditioned medium for 14 hours at 4 °C, using a FALC 205 rotator (FALC Instruments). Beads were then removed by centrifugation for 5 minutes at 1630 g. The supernatant was analyzed with a Bioplex-Luminex assay (Millipex® MAP Human Neurodegenerative Disease Magnetic Bead Panel 3, HNDG3MAG-36K; Millipore), to confirm BDNF depletion. Bioplex-Luminex assay was used a targeted proteomic analysis for BDNF and was performed according to the manufacturer’s guidelines. Samples were analyzed in a MAGPIX Luminex’s xMAP® instrument (Luminex Corporation, Texas, USA), and the protein concentration was calculated using the Bioplex ManagerTM 6.1 Software (Luminex Corporation, Texas, USA). The results are expressed in pg/ml.

### Immunocitochemistry

After stimulation, cells were subjected to a pre-fixation of 5 min in 1% paraformaldehyde (in phosphate buffered saline (PBS) with 4% sucrose^57^. Then the cells were fixed in 4% paraformaldehyde for 10 min at room temperature, washed three times with PBS and then permeabilized using PBS with 0.25% Triton X-100 for 5 min at room temperature. Next the cells were washed once with PBS followed by incubation in PBS with 3% bovine serum albumin (BSA), to block non-specific binding, for 30 min at room temperature. Primary antibodies were incubated overnight at 4 °C in PBS with 3% BSA. After incubation with the primary antibodies, the preparations were washed three times, 5 min each, with PBS to remove primary antibodies, then incubated with secondary antibodies in PBS with 3% BSA for 1 h at room temperature, and washed again twice, 5 min each, in PBS with 0.1% Triton X-100 and then washed in PBS for 5 min. Then, the glass slides mounted in prolong mounting media. The preparations were cured overnight at 4 °C protected from light, sealed with nailpolish and kept at 4 °C until microscopy analysis. The following antibodies were used: tau (chicken, Abcam) and β-tubulin (mouse, Sigma Aldrich), anti-chicken and anti-mouse Alexa-conjugated secondary antibodies 488 and 568, respectively (Life Technologies).

### Image acquisition and live-cell imaging

Imaging was performed either in a Zeiss Observer Z.1 microscope equipped with a Plan-Apochromat 20× air objective (0.8 numerical aperture); an AxioCam HRm camera and Zen Blue 2011 software. Experiments involving live cells were performed in imaging medium at 37 °C in a humidified atmosphere to avoid medium evaporation using a spinning disk confocal imaging system (CSU-X1M, Yokogawa) configured to a Zeiss Axio Observer Z1 microscope with a Plan-Apochromat 20× air objective (0.8 NA) coupled to an EM-CCD Evolve Delta camera and Zen Black 2012 Software. Images were obtained at 0 hours and 24 hours and neurons expressing Green Fluorescence Protein (~95% of the total population) were used to perform XY reconstructions of the somal and axonal compartments. Laser power was maintained to minimal levels to reduce phototoxicity. All conditions within the same experiment were processed in parallel and imaging settings (exposure time and laser power) were conserved during the entire experiment.

### Quantitative analysis

Individual neurons were randomly selected during acquisition. Axonal length and the longest axon segment of each neuron were measured with Image J Software.

Axonal network, present in the axonal compartment of microfluidic devices, of fixed cells stained with β-tubulin were quantified with Neurolucida software. This software measures the length of axonal segments present in each image, resulting in a final value corresponding to the sum of all axons present, i.e., to the total length of axonal network.

Quantification of the axonal outgrowth was performed using a MATLAB (The MathWorks Inc., Natick, MA) script named AxoFluidic^58^. This computational tool calculates the axonal networks density as a function of the distance (x-axis) to the microgrooves termination in the microfluidic chamber. The spatial scale of the axonal outgrowth is captured in a variable named lambda, λ, with units of micrometers. In the axonal elongation experiments lambda values from t = 24 hours where normalized to the lambda values from t = 0 hours. This ratio represents the axonal outgrowth occurred during those 24 hours, with respect to the basal outgrowth at t = 0 hours.

#### Individual axonal growth

Individual axons were randomly selected at 0 hours, and their length was measured with Image J Software. 24 h later the same axons were imaged and measured again. The values of axonal length of each axon were subtracted, according to the equation t_24_ − t_0_, obtaining the exact individual axonal growth during the 24 h stimulation period. These values were used to calculate the growth rate of each analyzed axon in the different conditions.

All images were processed and prepared for presentation using Photoshop and Illustrator (Adobe).

### Statistical Analysis

Results are presented as averaged values ± standard error of the mean (SEM). Graphs and statistical analysis were performed in Graph Pad Prism 5 Software. Statistical differences were examined by non-parametric tests (the test used for each experiment is indicated corresponding in the figure legend). Values of p < 0.05 were considered statistically significant (*p < 0.05; **p < 0.01, ***p < 0.001 and ****p < 0.0001).
